# Structural Design and Application of Desensitized FBG Force-Measuring Bolt

**DOI:** 10.3390/s22103930

**Published:** 2022-05-23

**Authors:** Minfu Liang, Yang Song, Xinqiu Fang, Yuye Jiang, Fan Zhang, Shuang Li, Ningning Chen, Ziyue Xu

**Affiliations:** 1 School of Mines, China University of Mining and Technology, Xuzhou 221116, China; liangmf2014@cumt.edu.cn (M.L.); ts21020199p21@cumt.edu.cn (F.Z.); ts15020009a3tm@cumt.edu.cn (N.C.); ts20020060a31@cumt.edu.cn (Z.X.); 2 Research Center of Intelligent Mining, China University of Mining and Technology, Xuzhou 221116, China; 3 School of Economics and Management, China University of Mining and Technology, Xuzhou 221116, China; lishuangchina@cumt.edu.cn; 4 Xuzhou Heavy Machinery Co., Ltd., Xuzhou 221116, China; jiangyuye20082009@126.com

**Keywords:** bolt supporting, force-measuring bolt, desensitization, fiber Bragg grating (FBG) sensors, drawing test

## Abstract

Bolt-supporting technology has been widely used in mine roadway support, and its own working conditions have important reference value for roadway safety support. In order to realize the continuous and reliable monitoring of the bolt rod’s working condition, this paper analyzes the existing problems of the existing fiber Bragg grating force-measuring bolt (FBG-FMB), and proposes a fiber grating strain desensitization sensing theory. Based on this theory, a desensitized FBG-FMB is developed with the spring as the elastic sensitive element. A mechanical analysis and drawing test show that the strain of the force-measuring bolt is greater than 60 times the micro-strain of the fiber grating, which verifies the feasibility of the structure design of the FBG-FMB. Finally, through the field application in the coal mine roadway, the working conditions of the bolt body at the two measuring points of the roadway are obtained to verify the reliability of the force-measuring bolt. In addition, the desensitized FBG-FMB can be widely used in the supporting fields of underground engineering such as slopes, tunnels, and foundation pits.

## 1. Introduction

As a simple active support method, the bolt-supporting technology has been widely studied and applied in various mine roadway support [1,2,3,4]. During the service life of the bolt-supported roadway, the real-time and accurate monitoring of its working conditions is of great significance to the normal maintenance of the roadway and the safe production of the mine.

At present, the axial force determination of the bolt can be divided into two categories according to its measuring principles: electrical and mechanical. The electrical measuring sensor can be further divided into resistance strain sensor and vibrating wire sensor. Both the resistance strain sensor and mechanical sensor measure the strain at different depths of the bolt, and then solve the stress through the relevant calculation method [5,6]. The stress is obtained by measuring the change in the vibration frequency of the steel string after the sensor is stressed at different depths [7,8]; However, these sensors have shortcomings such as being non-moisture-proof and non-waterproof, having poor anti-interference performance, and lacking the ability to achieve distributed measurement. Therefore, it cannot well meet the needs of real-time engineering monitoring. Furthermore, these sensors have a long detection period and cumbersome installation steps, which do not allow for effective long-term detection of bolt-supporting conditions.

The FBG sensor is an all-fiber passive device, which is a functional fiber sensor with FBG as a sensitive element. This type of sensor can be well adapted to the complex environment of coal mines and overcome the above problems [9,10,11,12,13,14,15]. At present, many scholars have carried out research on the application of FBG sensing technology to the monitoring of bolt conditions. Philipp M Nellen et al. [16] pioneered laboratory research on FBG-FMB. Martin Schroeck et al. [17] pasted the FBG on the surface of the bolt to monitor the working condition of the bolt. Jiang Desheng et al. [18] successfully applied the FBG stress sensing technology to the monitoring of bridge bolt cables. Chai Jing et al. [19] verified the feasibility of applying the FBG sensor to the force measuring of the bolt through the bolt pulling experiment. Liang Minfu et al. [20] designed the FBG Bolt Force Sensor by using cantilever beam and diaphragm as elastic components. Zhao Yiming et al. [21] studied the influence of groove shape on the strength of FBG-FMB, and provided practical suggestions for pasting glue in engineering practice. Wang Peng et al. [22] designed a full-length anchored FBG-FMB and monitoring system after deriving the conversion equation between the axial force of the bolt and the FBG wavelength variation. However, most of the existing FBG-FMB have the problem that the strain of the bolt and the FBG strain are uncoordinated, so the FBG is very easy to break in the field application, which greatly reduces the reliability of the FBG-FMB.

Based on the FBG-FMB practically applied in coal mine roadway, this paper proposes a desensitized FBG-FMB and introduces its monitoring method. The feasibility and reliability of the sensor are verified through mechanical analysis, pull-out experiments and field applications. The sensor solves the problem of the uncoordinated strain between the grating and the bolt, which can realize the early warning of the failure of the bolt and accumulate the original data for the further optimization of the roadway support parameters. The force-measuring bolt and its monitoring system have great application prospects in the field of coal mine roadway support, and have wide promotion value in engineering fields such as slopes, tunnels, and foundation pits.

## 2. Main Problems of Current FBG-FMB

### 2.1. Structure and Defects of Current FBG-FMB

**Figure 1:**
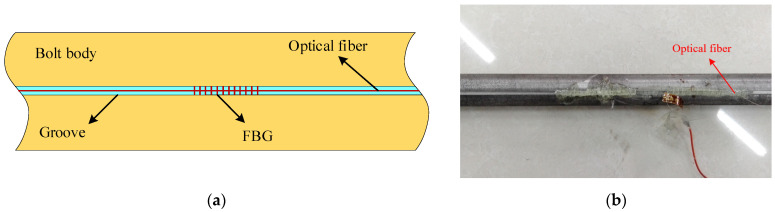
Structure schematic and photo of the existing FBG-FMB for laboratory test. (**a**) Structure schematic; (**b**) FBG-FMB Photo.

The current FBG-FMB usually has a groove (2 mm wide and 1 mm deep) on the bolt rod body for attaching fiber gratings. The structure of the force-measuring bolt is shown in Figure 1. When the axial strain occurs in the force-measuring bolt, the FBG pasted in the groove will generate strain along with it. Then, demodulate and analyze the signal transmitted by the FBG to achieve the purpose of accurately measuring the axial strain of the force-measuring bolt [23,24].

However, through theoretical analysis and experimental tests, there is a problem of mismatch between the FBG elongation and the force-measuring bolt in the design of the sensing structure.

The sensing principle of the FBG-FMB is that when the bolt is subjected to a strain, the FBG attached to the rod generates strain along with it. In order to achieve accurate measurement of strain, it is required that the strain generated by the force-measuring bolt and the FBG be as consistent as possible. The maximum elongation of force-measuring bolt in practice is 17%, while the FBG is 0.3%. Once the elongation of the force-measuring bolt is greater than 0.3%, it will not only fail to achieve the purpose of force measurement, but also lead to damage of the fiber grating.

### 2.2. Coordination Analysis of Current FBG-FMB

Through the analysis of the experimental data, it can be proved that the fracture of the FBG-FMB may break due to the uncoordinated strain between the grating and the bolt.

**Figure 2:**
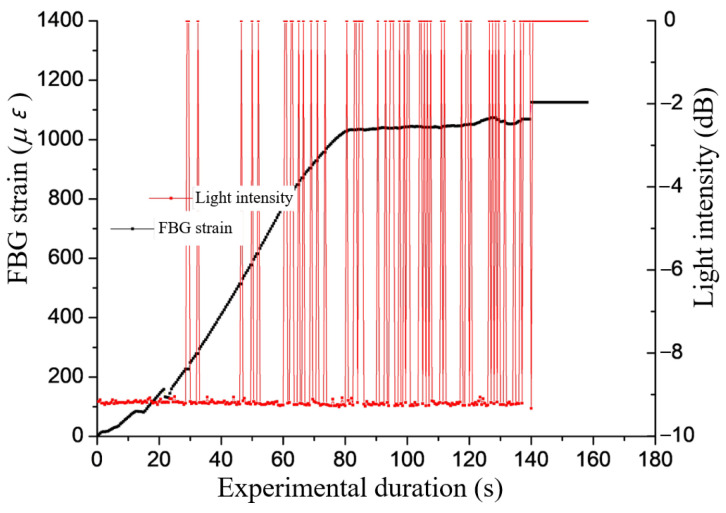
Variation of FBG strain and light intensity with time.

As can be seen from Figure 2, the grating breaks at around 18 s where the red and black lines intersect. However, at this time, the force-measuring bolt is still in the elastic stage and is far from reaching the yield limit. Therefore, if the tensile force on the force-measuring bolt is greater than that when the grating is broken, the grating will be broken, and the force-measuring effect will be lost.

**Figure 3:**
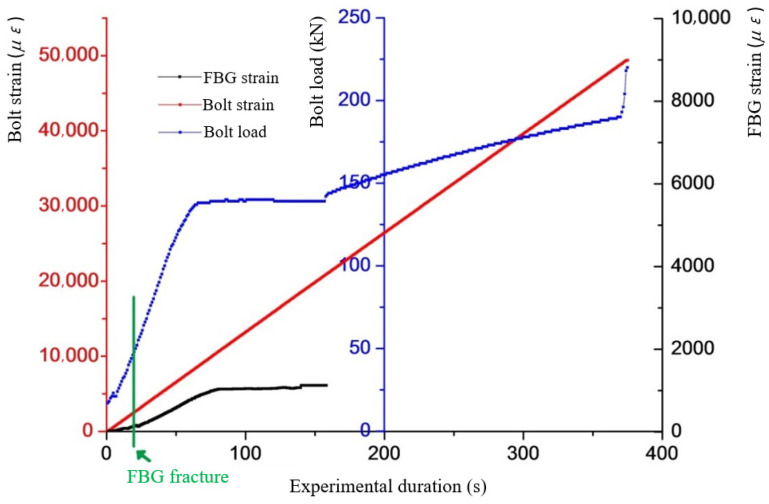
Variation diagram of bolt strain and load and FBG strain with time.

As can be seen from Figure 3, the load on the bolt rod when the grating is broken is approximately 50 kN. Therefore, when the load is greater than 50 kN, the grating will lose its force measurement effect because it is pulled off. So, it is essential to develop the structure of the desensitized FBG-FMB.

## 3. Sensing Principle of Desensitized FBG-FMB

### 3.1. Basic Principles of FBG Sensing

**Figure 4:**
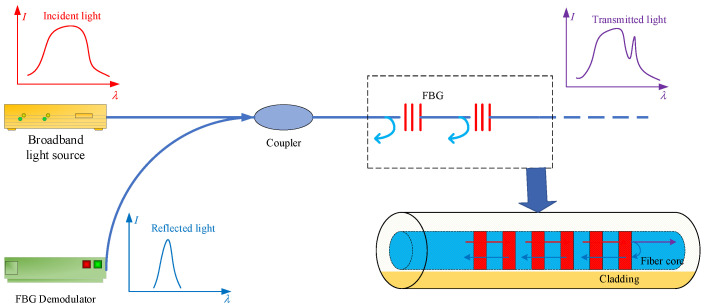
FBG sensing schematic.

FBG is a grating with grids inside the fiber core that can be formed by inscribing the fiber by methods such as holographic or phase masking. Thus, the refractive index of the fiber core changes periodically, resulting in regular changes in both the transmission spectrum and the reflection spectrum when the light wave transmits in the fiber grating. The principle of FBG sensing is shown in Figure 4.

According to Maxwell’s equations and coupled-mode theory for optical fiber [25], when a beam of light transmits in a fiber and satisfies the Bragg grating condition, the light will be effectively reflected. At this time, the peak of the reflection spectrum is the fiber Bragg wavelength. In a single-mode fiber, the basic expression for the Bragg center wavelength is expressed as follows:

(1) $$\lambda_{B}=2n_{eff}\Lambda ,$$

where *λ_B_* is peak wavelength of reflection spectrum, *n_eff_* effective refractive index of fiber core, and Λ is the grating period.

FBG has temperature-strain cross-sensitive characteristic. Changes in either of these two factors result in changes in its effective refractive index and grating period, which in turn cause changes in the reflected wavelength. The relationship between the amount of FBG wavelength change and temperature and strain is expressed as follows [26]:

(2) $$\frac{\Delta \lambda_{B}}{\lambda_{B}}=\left(1-p_{e}\right)\varepsilon_{z}+\left(\alpha +\xi \right)\Delta T,$$

where Δ*λ* is the wavelength variation of FBG, *p_e_* is effective elasto-optical coefficient, *α* and *ξ* are thermal expansion coefficient and thermal-optic coefficient of fiber, respectively, *ε_z_* is axial strain of fiber grating, Δ*T* is temperature variation.

When the fiber grating is not affected by temperature but only by stress, the change in the center wavelength of the reflected light is positively correlated with the axial strain on the fiber [27]:

(3) $$\frac{\Delta \lambda_{B}}{\lambda_{B}}=K_{\varepsilon }\varepsilon_{z},$$

where *K_ε_* = 1 − *p_e_* is sensitivity coefficient.

### 3.2. Desensitized Strain Sensing Principle

**Figure 5:**
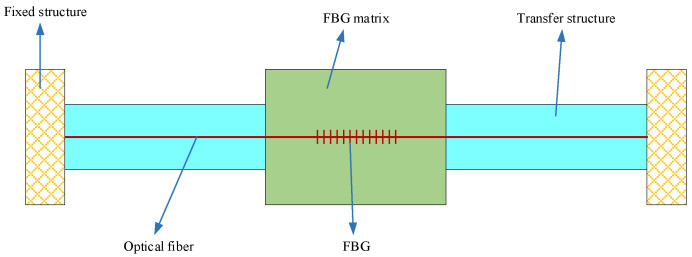
Schematic diagram of FBG strain desensitization sensor.

In order to realize the FBG strain desensitization sensing, the strain of the FBG matrix should be as small as possible, while transmission structure is the opposite. The principle of FBG strain desensitization sensing is shown in Figure 5. The middle part in the figure represents the FBG matrix. The FBG is attached to the matrix surface along the axial direction. Both ends are represented as fixed structures, which are fixed on the bolt. When the bolt is stressed, the tensile force is transmitted to the matrix through the fixed structure at both ends and the transmission structure to generate strain. Finally, the FBG pasted on the matrix is strained.

According to the theory of material mechanics [28], the deformation quantities of the transfer structure (Equation (4)) and the FBG matrix (Equation (5)) are expressed as follows:

(4) $$\Delta L_{s}=\frac{P_{s}L_{s}}{E_{s}A_{s}},$$

(5) $$\Delta L_{f}=\frac{P_{f}L_{f}}{E_{f}A_{f}},$$

where *L_s_* is the axial force length of the transfer structure, Δ*L_s_* is the strain variation of transfer structure, *E_s_* is the transfer structural elastic modulus, *A_s_* is the transfer structure cross-sectional area, *L_f_* is the axial force length of the FBG, Δ*L_f_* is the strain variation of the FBG, *E_f_* is the FBG elastic modulus, and *A_f_* is the FBG cross-sectional area.

Since the transfer structure and the FBG matrix are a whole, it can be obtained that *P_s_* = *P_f_*. Combining Equations (4) and (5) together, we can obtain:

(6) $$\frac{\varepsilon_{s}}{\varepsilon_{f}}=\frac{E_{f}A_{f}}{E_{s}A_{s}},$$

where *ε_s_* is the transfer structural strain, *ε_f_* is the strain of FBG matrix.

Simplify Equation (6) as follows:

(7) $$\varepsilon_{f}=K\varepsilon_{s},$$

in the formula, $K=\frac{E_{s}A_{s}}{E_{f}A_{f}}$. In order to achieve the purpose of desensitization, the axial strain of the FBG matrix needs to be smaller than the axial strain of the transfer structure. It can be expressed as *K* < 1. Therefore, it is necessary to find a material with a relatively large elastic modulus *E_f_* and a large cross-sectional area *A_f_* as the FBG matrix, while the transfer structure is just the opposite.

## 4. Design and Test of Desensitized FBG-FMB

### 4.1. Structural Design of Desensitized FBG-FMB

In this paper, a spring-desensitized FBG-FMB is designed, which not only has the advantages of being moisture-proof, waterproof and anti-interference, but can also solve the problem that the strain of the fiber grating and the strain of the bolt are not synchronized.

**Figure 6:**
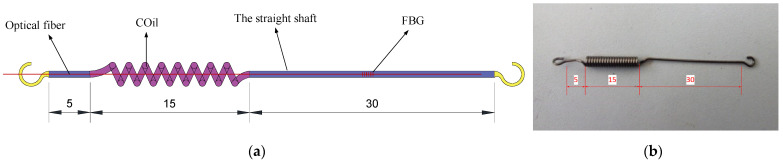
The structure and photo of spring desensitization sensor. (**a**) Structure diagram; (**b**) spring photo.

The spring-desensitized sensor is the core element in the design of the desensitized FBG-FMB. The material in the desensitization structure is mainly a custom-made spring, as shown in Figure 6. The FBG is bonded to the straight shaft part of the spring by adhesive, and passes through the center when the spring coil part. Finally, the spring is embedded in the sensor groove of the bolt body.

**Figure 7:**
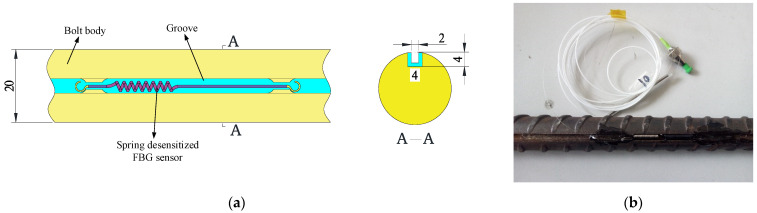
Schematic diagram and photo of the bolt structure where the sensor is located. (**a**) Schematic diagram; (**b**) photo.

The design of the force-measuring bolt body is first to produce a groove on the surface of the bolt to accommodate the FBG that transmits the signal. The groove is 4 mm square and runs through the entire bolt body. In addition, three positions in the slot are selected to design the caulking groove to fix the spring-desensitized sensor. A partial plan and profile of the spring-desensitized sensor groove are shown in Figure 7.

### 4.2. Mechanical Analysis of Spring-Desensitized Sensor

The spring-desensitized sensor mounted on the bolt rod will also stretch as the bolt rod is stretched, but the strain generated by the stretched spring is mainly concentrated at the spring helix. At this time, the strain generated by the matrix where the fiber grating is pasted will be relatively small, thus protecting the fiber grating from prematurely breaking during the stressing process of the bolt rod. The material of the spring used for the sensor is piano steel wire, and its main parameters are shown in Table 1.

**Figure 8:**
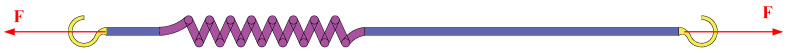
Force analysis of spring-desensitized sensor.

When the desensitized FBG-FMB is pulled, the force analysis of the spring-desensitized sensor is shown in Figure 8. According to the theory of elasticity and material mechanics [28,29], the deformation of the helical part of the spring (Equation (8)) and the straight shaft part of the spring (Equation (9)) are expressed as follows:

(8) $$\Delta x_{1}=\frac{F}{k},$$

(9) $$\Delta x_{2}=\frac{FL}{EA},$$

where Δ*x*_1_ is the deformation of the helical part of the spring, *F* is the axial force on the spring-desensitized sensor, *k* is the elastic coefficient of the spring, Δ*x*_2_ is the deformation of the straight shaft part of the spring, *L* is the length of the straight shaft of the spring, *E* is the elastic modulus of the straight shaft part of the spring, *A* is the cross-sectional area of the straight shaft portion of the spring, and *A* = *πr*^2^, *r* is the straight shaft radius of the spring.

Substitute the data in Table 1 into Equations (8) and (9), the result is as follows:

(10) $$\frac{\Delta x_{1}}{\Delta x_{2}}=330,$$

In addition, the elongation of the fiber and steel is 0.3% and 17%, respectively. The ratio of the two deformations is 60. However, when the sensor is under stress, the ratio of the deformation amount of the spring helical part to the straight shaft part is 330. Comparing the two, the deformation value 330 is significantly greater than 60. Thus, it can ensure that the fiber is not pulled off.

The force measurement accuracy of FBG is 300 micro-strains, which is expressed as follows:

(11) $$\frac{\Delta x_{2}}{L}=\frac{F}{EA}\;>\;300\times 10^{-6},$$

In summary, the data can be measured when the force of the force-measuring bolt is greater than 23.1 N.

### 4.3. The Desensitized FBG-FMB Test

**Figure 9:**
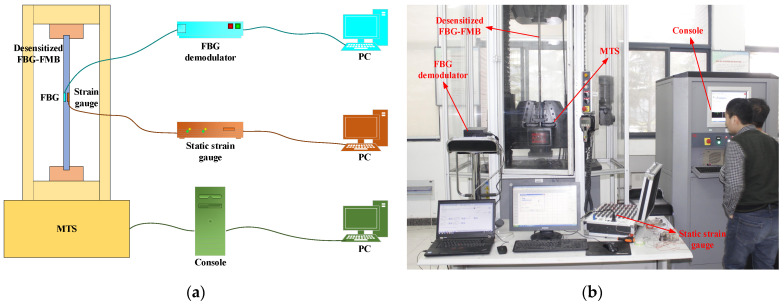
Experimental system principle picture and photo. (**a**) Experimental schematic diagram; (**b**) photo of experimental system.

The bolt test system is shown in Figure 9. The experimental equipment mainly includes MTS testing machine, SM125 fiber grating demodulator and TS3866 static resistance strain gauge. In the experiment, the test bolt is not only equipped with a spring-desensitized FBG sensor, but also with strain gauges attached to its surface. The test bolt is loaded by the MTS testing machine and the loading process is controlled by the testing machine console. The spring-desensitized fiber grating sensor on the test bolt is connected to the FBG demodulator through an optical fiber, and the demodulator is connected to the PC through a network cable. The strain gauges on the test bolt are connected to the static resistance strain gauge through the wire, and the strain gauge is connected to the PC through the network cable.

In this experiment, bolt strain and fiber grating strain values are recorded and plotted for comparison by drawing bolt rod.

**Figure 10:**
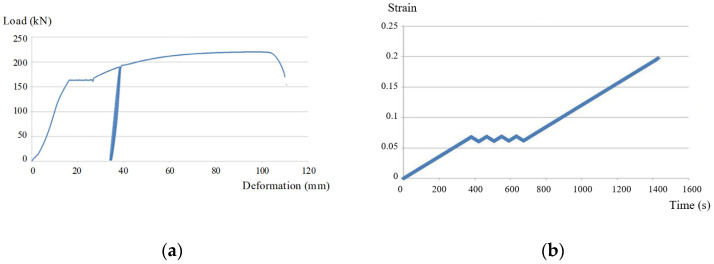

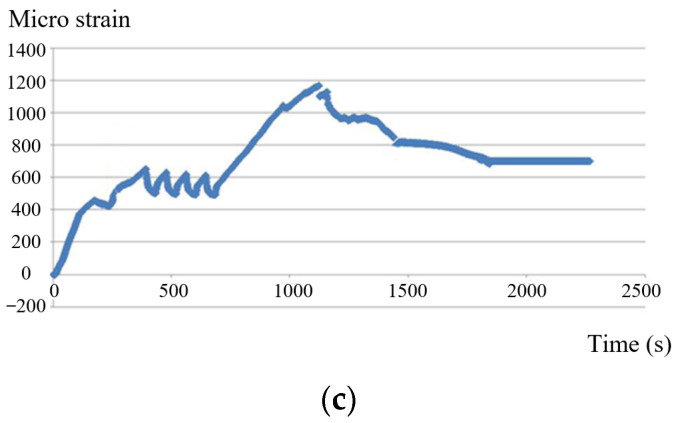
Variation diagram of loading, bolt strain and FBG micro-strain with time. (**a**) The loading diagram of the bolt; (**b**) bolt strain diagram; (**c**) FBG micro-strain diagram.

The deformation state of the anchor rod during tension can be seen from Figure 10a. The drawing test shows that the deformation variable of the desensitized FBG-FMB first increases with a certain slope with the increase in load, and then remains almost unchanged for a small section, and then the slope of the load elongation curve gradually decreases, which means that the deformation change corresponding to the same load change is expanded, that is, the deformation change increases with the increase in load. After the slope decreases to zero, the load required to cause deformation begins to decrease until the bolt fracture shows a vertical decrease in slope.

Comprehensive analysis of Figure 10b,c shows the desensitization effect of desensitized FBG-FMB. From Figure 10b, it can be seen that the bolt strain shows a linear upward trend after 650 s. The stress change in the bolt from 650 s to 1000 s is about 0.06~0.12. From Figure 10c, it can be seen that the FBG strain is linear between 850 s and 1000 s, which is consistent with the bolt strain trend. The micro-strain of the FBG from 850 s to 1000 s is about 600~1000. In conclusion, the strain of the bolt is 60 times greater than the micro-strain of the FBG, which is consistent with the mechanical analysis in Section 4.2.

## 5. Field Test and Results Analysis

**Figure 11:**
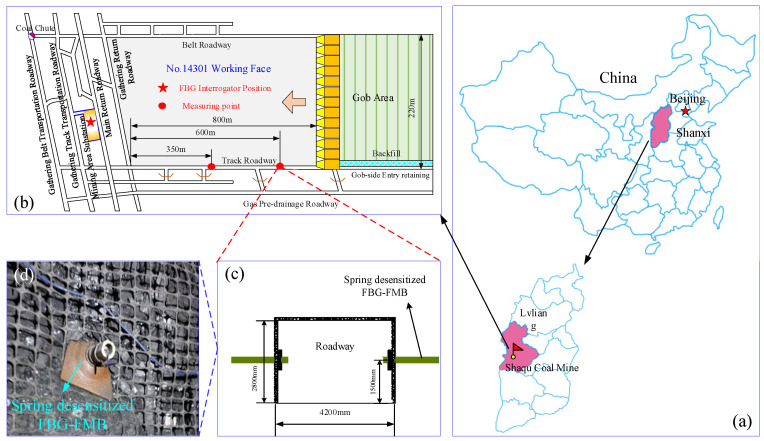
The location of No.14301 working face and bolt arrangements in Shaqu Mine. (**a**) Location of Shaqu Coal Mine; (**b**) plan view of No.14301 working face; (**c**) layout position of bolts; (**d**) photo of bolt arrangement in roadway.

The desensitized FBG-FMB is tested in the field at the No.14301 long wall mining face along strike of Shaqu Coal Mine (Lvliang City, Shanxi Province, China, as shown in Figure 11a). Field tests are carried out to verify the reliability of the bolt and realize its engineering application.

### 5.1. Project Overview and Monitoring Plan

#### 5.1.1. No.14301 Working Face Project Overview

The working face is buried 400 m deep with an average inclination angle of 4°. It has a length of 1145 m and a width of 220 m, with a vertical stress of approximately 10 Mpa. Its gas content can reach 81.84 m^3^/t, and it is identified as a high gas mine.

#### 5.1.2. Track Lane Monitoring Scheme

##### Arrangement Principle of Desensitized FBG-FMB

1. The number of FBG-FMB to be placed needs to comprehensively consider the engineering geological conditions, mining conditions, construction characteristics and other influencing factors of the No.14301 working face.
2. Bolt monitoring points should first be considered for placement at key stress points in the roadway.
3. When selecting the position of the bolts arrangements at the monitoring points, it is necessary to consider the construction conditions and difficulty in the roadway.
4. When arranging the bolts, the normal stress condition of the surrounding rock structure of the roadway should be affected as little as possible.

##### Monitoring Points Layout

When the monitoring system is installed, the working face has been mined to 800 m from the roadway entrance. The FBG signal demodulation host is placed at the third mining area substation to the south. Considering the location of the working face and the position of the stopping line, it is decided to set up two measuring points in the No.14301 track lane. The first and second measuring points are arranged at 350 m and 600 m from the entrance of the No.14301 track lane, respectively, as shown in Figure 11b.

##### Layout of Desensitized FBG-FMB

**Figure 12:**
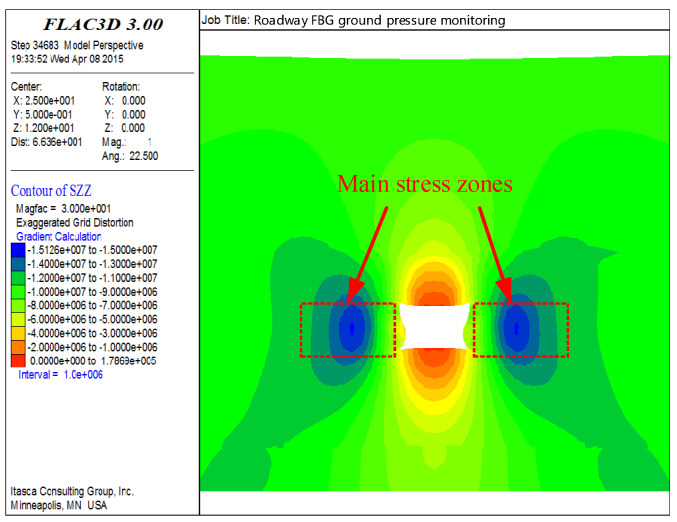
Stress and displacement distribution of surrounding rock.

Under the condition of static stress, the key stress points in the surrounding rock structure of the roadway are selected to arrange the FBG-FMB. Combined with the overburden mechanical parameters of No.14301 working face, the excavation state of the roadway is analyzed by numerical simulation software. Finally, the stress and displacement distribution diagram of surrounding rock can be obtained, as shown in Figure 12.

From stress and displacement distribution of the surrounding rock in Figure 12, it can be seen that the surrounding rock stress is mainly concentrated on the two sides of the roadway. Therefore, two sides of each monitoring point are provided with one desensitized FBG-FMB. The bolts used for monitoring are all 20 mm in diameter and 2000 mm in length. They are installed at a height of 1.5 m from the bottom plate. The bolts installation arrangements are shown in Figure 11c,d.

### 5.2. Analysis of Monitoring Results

**Figure 13:**
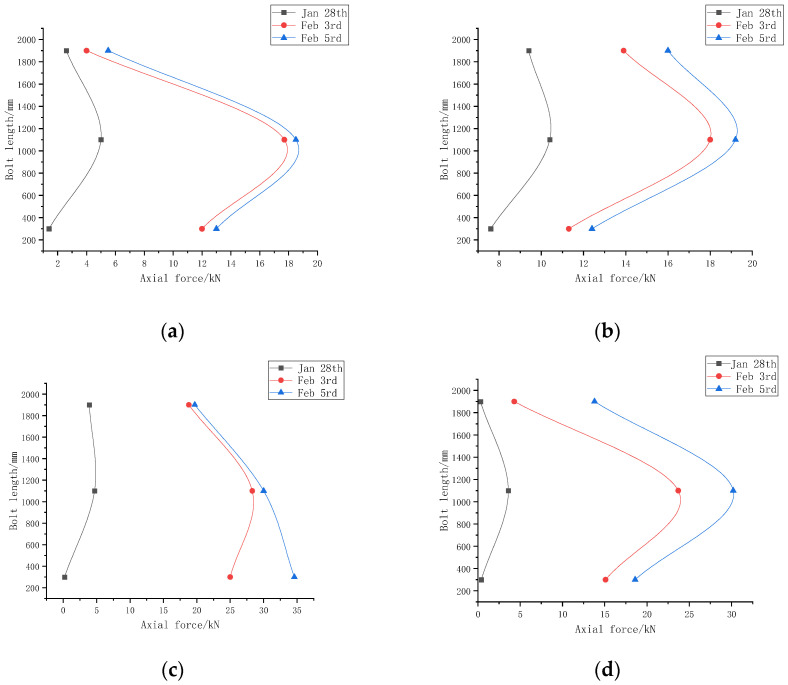
Distribution of the axial stress of the bolt along the body at two measuring points. (**a**) Axial stress distribution diagram of left bolt at No. 1 measuring point; (**b**) Axial stress distribution diagram of right bolt at No. 1 measuring point; (**c**) Axial stress distribution diagram of left bolt at No. 2 measuring point; (**d**) Axial stress distribution diagram of right bolt at No. 2 measuring point.

The selected monitoring points are continuously monitored for 7 days, during which the temperature of the monitoring points is basically constant. Thus, the effect of temperature on the sensor can be ignored in the subsequent data processing and experimental analysis. So, the subsequent analysis can approximately think that the wavelength shift in the FBG in the sensor is only caused by the change in the bolt force. The experimental data of three days are selected to sort and plot the corresponding axial stress distribution of the anchors, as shown in Figure 13. Among them, the location of the working face is 783 m from the return air roadway on 28 January; additionally, on 3 and 5 February, the working face position is 768 m and 762 m away from the return air roadway, respectively.

As shown in Figure 13, the overall axial stress of the force-measuring bolt is larger in the middle and smaller at the ends. As the working face continues to advance, the axial stress of the bolts at both monitoring points is increasing. During the period from 28 January to 3 February, the stress changes abruptly, but the bolts conditions still perform well. When the working face is 762 m away from the return air roadway, the left side force-measuring bolt of the No. 2 measuring point is greatly affected by the mining of the working face. At this time, the axial stress of the bolt is significantly increased, but it is still in the safe range. The field test of the track roadway in the No.14301 working face of Shaqu Coal Mine shows that the force-measuring bolt can stably, continuously and accurately measure the mine pressure of the roadway and monitor the bolt support conditions.

## 6. Conclusions

(1) Experiments on current FBG-FMB show that the fiber in the bolt will be broken when the elongation of the bolt is greater than 0.3%. Additionally, at 50 kN or more, the fiber will be pulled off and lose its sensing effectiveness. This shows that there is a problem of mismatch between FBG elongation and force-measuring bolt.
(2) In order to solve the problems in the existing FBG force-measuring bolt, a FBG strain desensitization sensing model is established based on the principle of FBG sensing and material mechanics. Then, a spring-desensitized FBG sensor is designed. Through mechanical analysis and experimental verification, the sensor successfully solves the problem of the uncoordinated strain of the bolt and the fiber.
(3) In the field test of the track roadway of the No.14301 working face in Shaqu Coal Mine, the axial stress of the spring-desensitized FBG-FMB is larger in the middle and smaller in the end. The axial stress of the bolts at the monitoring point increases with the advancement of the working face, and increases more sharply when affected by the mining of the working face. This application shows that the force-measuring bolt has good reliability and field applicability.
(4) This study has potential limitations due to workload and other reasons. The desensitized FBG-FMB may still be affected by the change in ambient temperature in use, and the sensor can still be improved in temperature compensation; additionally, the desensitized FBG-FMB lacks supporting data processing system, and the bolt state visualization needs to be further studied.

## Figures and Tables

**Table 1:** Characteristics of spring-desensitized sensors.

Parameter	Symbol	Numerical Value
elasticity coefficient	*k*	7.84 × 103 N/m
straight shaft length	*L*	30 mm
elastic modulus	*E*	200 GPa
straight shaft radius	*r*	0.35 mm

## Data Availability

All data and code used or analyzed in this study are available from the corresponding author on reasonable request.
